# Quality of flow diagram in systematic review and/or meta-analysis

**DOI:** 10.1371/journal.pone.0195955

**Published:** 2018-06-27

**Authors:** Hai Vu-Ngoc, Sameh Samir Elawady, Ghaleb Muhammad Mehyar, Amr Hesham Abdelhamid, Omar Mohamed Mattar, Oday Halhouli, Nguyen Lam Vuong, Citra Dewi Mohd Ali, Ummu Helma Hassan, Nguyen Dang Kien, Kenji Hirayama, Nguyen Tien Huy

**Affiliations:** 1 Faculty of Medicine, University of Medicine and Pharmacy, Ho Chi Minh City, Vietnam; 2 Faculty of Medicine, Tanta University, Tanta, Egypt; 3 Al-Essra Hospital, Amman, Jordan; 4 Harvard Medical School, Boston, Massachusetts, United States of America; 5 Menoufia University Hospitals, Menoufia, Egypt; 6 Kasr Al Ainy School of Medicine, Cairo University, Cairo, Egypt; 7 Faculty of Medicine, University of Jordan, Amman, Jordan; 8 University of Medicine and Pharmacy, Ho Chi Minh City, Vietnam; 9 Department of Obstetrics and Gynecology, Thai Binh University of Medicine and Pharmacy, Thai Binh, Vietnam; 10 Department of Immunogenetics, Institute of Tropical Medicine (NEKKEN), Leading Graduate School Program, and Graduate School of Biomedical Sciences, Nagasaki University, Nagasaki, Japan; 11 Evidence Based Medicine Research Group & Faculty of Applied Sciences, Ton Duc Thang University, Ho Chi Minh City, Vietnam; 12 Department of Clinical Product Development, Institute of Tropical Medicine (NEKKEN), Leading Graduate School Program, and Graduate School of Biomedical Sciences, Nagasaki University, Nagasaki, Japan; Ottawa Hospital Research Institute, CANADA

## Abstract

Systematic reviews and/or meta-analyses generally provide the best evidence for medical research. Authors are recommended to use flow diagrams to present the review process, allowing for better understanding among readers. However, no studies as of yet have assessed the quality of flow diagrams in systematic review/meta-analyses. Our study aims to evaluate the quality of systematic review/meta-analyses over a period of ten years, by assessing the quality of the flow diagrams, and the correlation to the methodological quality. Two hundred articles of “systematic review” and/or “meta-analysis” from January 2004 to August 2015 were randomly retrieved in Pubmed to be assessed for the flow diagram and methodological qualities. The flow diagrams were evaluated using a 16-grade scale corresponding to the four stages of PRISMA flow diagram. It composes four parts: Identification, Screening, Eligibility and Inclusion. Of the 200 articles screened, 154 articles were included and were assessed with AMSTAR checklist. Among them, 78 articles (50.6%) had the flow diagram. Over ten years, the proportion of papers with flow diagram available had been increasing significantly with regression coefficient beta = 5.649 (p = 0.002). However, the improvement in quality of the flow diagram increased slightly but not significantly (regression coefficient beta = 0.177, p = 0.133). Our analysis showed high variation in the proportion of articles that reported flow diagram components. The lowest proportions were 1% for reporting methods of duplicates removal in screening phase, followed by 6% for manual search in identification phase, 22% for number of studies for each specific/subgroup analysis, 27% for number of articles retrieved from each database, and 31% for number of studies included in qualitative analysis. The flow diagram quality was correlated with the methodological quality with the Pearson’s coefficient r = 0.32 (p = 0.0039). Therefore, this review suggests that the reporting quality of flow diagram is less satisfactory, hence not maximizing the potential benefit of the flow diagrams. A guideline with standardized flow diagram is recommended to improve the quality of systematic reviews, and to enable better reader comprehension of the review process.

## Introduction

Systematic review is a form of literature review that assembles and analyzes several studies related to a specific question, with the aim of synthesizing the respective findings of the studies, basing on the methods framed at the beginning of the procedure [1–4]. It may include a meta-analysis (a quantitative synthesis) depending on the available data [5,6], and provides one of the best evidence of medical research, hence providing useful clinical data for decision making in actual practice [7–10]. Further, systematic reviews detect gaps in the literature review of the specific topic, which can be a springboard for future research to address these gaps. [11–13].

A systematic review is started with a meticulous search of the literature for relevant papers. First, all databases and citation indexes searched, such as Web of Science, Embase, or PubMed, as well as any hand-searched individual papers will be carefully recorded in the methodology section of the manuscript. In the next step, the titles and/or abstracts of the identified articles are screened with respect to the determined inclusion and exclusion criteria for eligibility and inclusion. The methodological quality of each included study may be objectively assessed using the high quality standards of Cochrane collaboration [8], or other quality assessment tools. Flow diagrams allow readers to have a general idea of the process flow via a single glance at the numbers, brief words and direction of arrows, and is hence very important and useful in a review—which demands a long procedure of gathering original papers. A high-quality flow diagram can help readers or reviewers assess logical stages of the process as well as define the boundaries of the process. A high-quality flow diagram must contain full items for all four stages based on the PRISMA flow diagram (S1 Fig). Besides, the adherence to PRISMA checklist should be evaluated for all included systematic reviews and/or meta-analysis [14,15].

The work of Egger and colleagues demonstrated the need for the evaluation of CONSORT flow diagram. Although it is not about systematic reviews, it can be considered as related literature to our work [16]. A previous study revealed that only 49% of the systematic review articles illustrated the study selection process with a diagram. [17] Another study assessed the quality of systematic review/meta-analysis based on the abstract [18]. And Page et al reported in 2016 on flow diagrams in reports of systematic reviews, they considered whether the diagram was fully presented, partially, or not at all, although did not give much detail [19]. Few studies as of date, have provided a detailed assessment of the reporting quality of flow diagrams in systematic review/meta-analysis. Therefore, we aimed to: (i) study the proportion of flow diagram existing in systematic review articles, (ii) evaluate the quality of flow diagrams, and (iii) determine the association between the quality of flow diagram and the methodological quality of the systematic review/meta-analysis measured by AMSTAR score.

## Materials and methods

### Selection of articles

Firstly, we searched for systematic reviews or meta-analyses in Pubmed from January 2004 to August 2015 with the search term: (“systematic review”[All Fields] OR “systematic literature review”[All Fields]) OR “meta-analysis”[All Fields] AND (“2004/01/01”[PDAT]: “2016/12/31”[PDAT]). Then, 200 articles were randomly retrieved according to 200 random numbers generated on August 16^th^ 2015 at 19:24:18 by Random Number Generator of website http://www.psychicscience.org/random.aspx, and were subsequently screened. We included systematic reviews with or without meta-analysis. We excluded any narrative review, case report, protocol, editorial, or book chapter in addition to any duplicated articles. We made no restriction on publication language, given the potential influence of publication language on results [20,21]. Then, the included articles were assessed by the methodological quality (with AMSTAR score checklist containing 11 points) and the flow diagram quality [22].

### Assessment of methodological quality

The methodological quality of each included systematic review and/or meta-analysis was assessed through the 11-grade AMSTAR checklist for its advancement, reliability and ease to use for evaluating systematic review [23,24]. Both internal and external validations of the AMSTAR tool have been reported [23,25]. The checklist consists of 11 questions, corresponding to 11 points (S1 Table) [22]. A Measurement Tool to assess Systematic reviews (AMSTAR) is an instrument used in assessing the methodological quality of systematic reviews, and also acts as a guide to conduct reviews [22]. The adherence of each among a total of 27 items of PRISMA checklist were also extracted and evaluated by two independent authors (OH, GMM). In case of any discrepancy between two authors, the final decision was reached by consensus of the project’s supervisor (NTH). Then the items were reported as percentages (NLV, VNH).

### Quality assessment of flow diagram

Basing on PRISMA flow diagram, we developed a 16-grade scale which is composed of four parts, corresponding to four key processes of the flow diagram: Identification, Screening, Eligibility and Inclusion [26,27]. The score was counted as value “1” (for the answer “Yes”) and “0” (for the answer “No). Flow diagram score was calculated by summing all the points based on the 16-grade scale (S2 Table). The AMSTAR score and flow diagram score were evaluated by three independent reviewers. When disagreement occurred, a consensus decision was made following discussion with senior-reviewers (NTH, KH). The flow diagram score was then compared between Cochrane and non-Cochrane reports including flow diagrams.

### Data analysis

Statistical software R version 3.2.3 was used for our data analysis. (http://www.r-project.org/). 2-tailed Student t-test was used to compare the flow diagram score and AMSTAR scores between different groups. These included comparing articles with and without flow diagram, as well as articles published before 2009 and after 2009, when PRISMA was introduced. Pearson’s correlation coefficient and scatter plot were further conducted to evaluate the correlation between flow diagram and AMSTAR score. Since impact factor is not normally distributed, Spearman’s correlation coefficient was used to evaluate the correlation between flow diagram score and impact factor of journals. All analyses were considered statistically significant if the P-value < 0.05. Regression analysis was used to reveal the trend of flow diagram score (quality of flow diagram) and AMSTAR score (methodological quality of systematic reviews) over a period of ten years. Scatter plots were used to show estimates of how methodological quality of systematic reviews and how quality of flow diagrams relates to year of publication.

## Results

**Fig 1:**
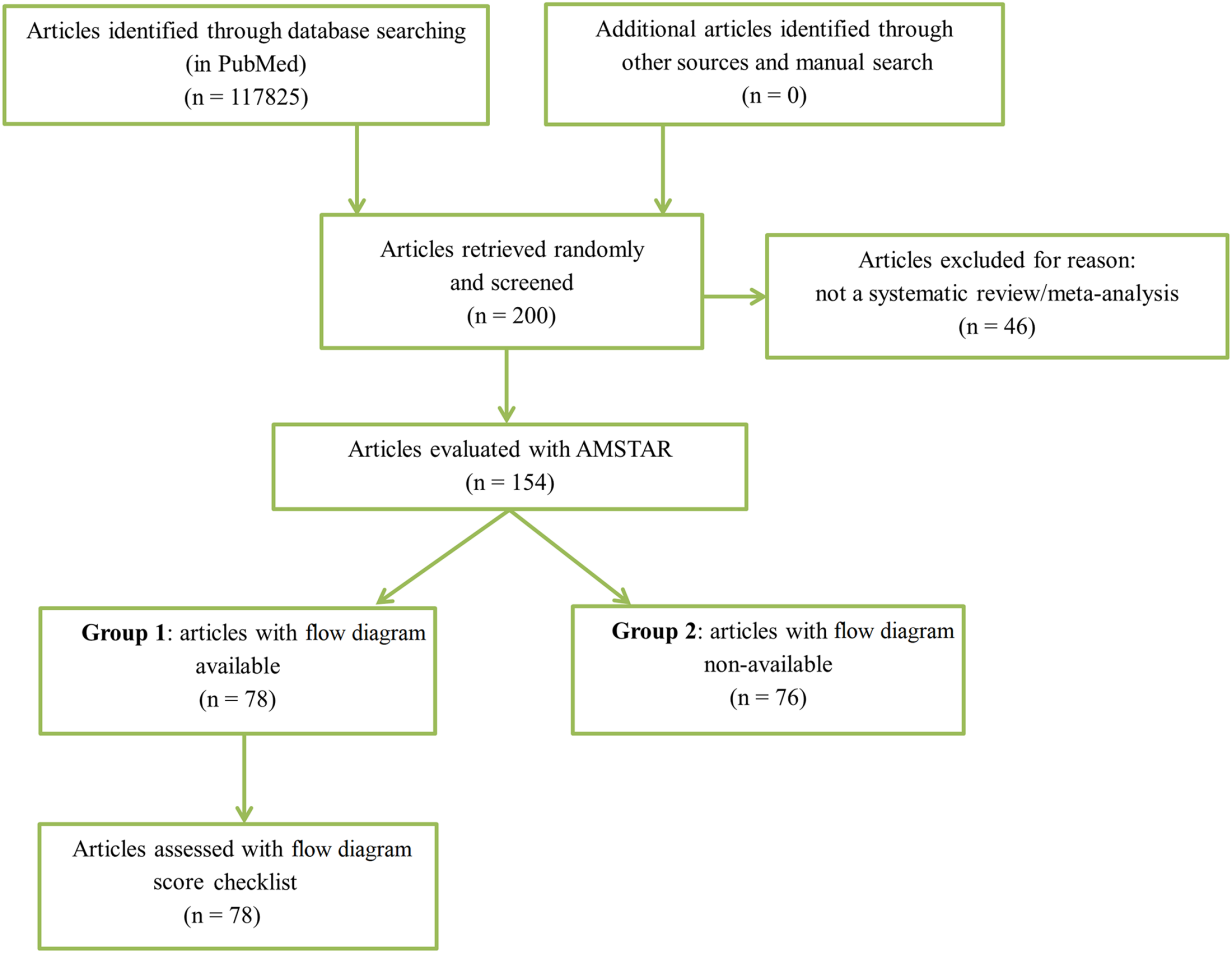
Diagram of selecting systematic reviews for the study.

From January 2004 to August 2015, we found 117825 articles with the search term above. Among 200 randomly chosen articles, 154 systematic reviews and/or meta-analysis met our inclusion criteria (Fig 1).

### Proportion of systematic review articles presenting flow diagram

**Fig 2:**
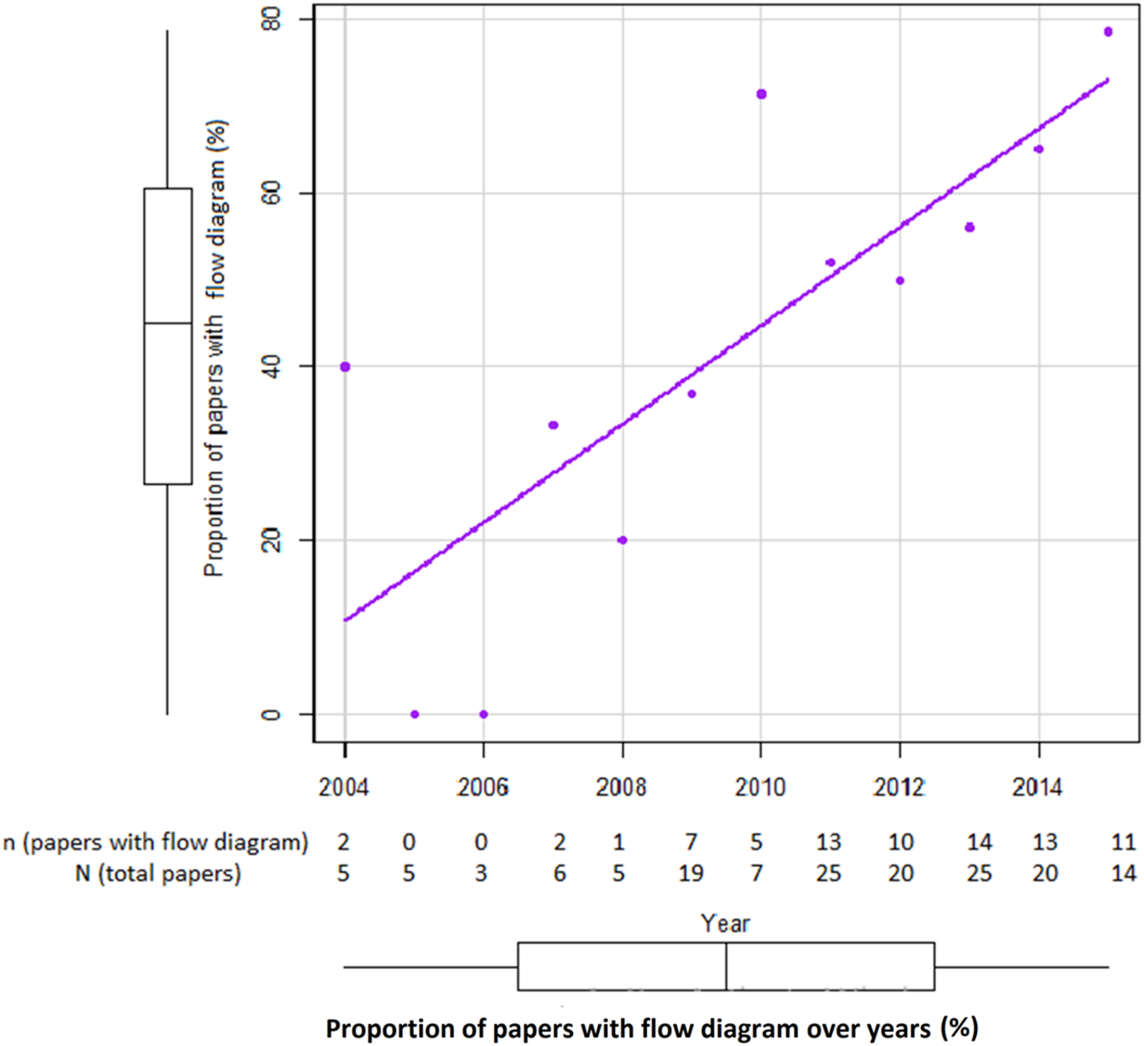
The proportion of papers with flow diagram over ten years.

Of those 154 included systematic reviews and/or meta-analyses, there are 78 papers (51%) with flow diagrams and 76 papers (49%) without flow diagrams. Of the 78 papers with flow diagram, 66 papers (85%) were published after 2009 when PRISMA statement was declared [14,26]. The frequency of flow diagrams in systematic review/meta-analysis before and in 2009, and after 2009 was 28% (12 out of 43 papers) and 59% (66 out of 111 papers), respectively. Over ten years, the proportion of papers with flow diagram available had been significantly increasing with the Pearson correlation r = 0.80 (p = 0.002) and the regression coefficient beta = 5.649 (p = 0.002), when weighted for number of SR/MA in each year, the regression coefficient beta = 5.290 (p = 0.0007) (Fig 2).

### Quality assessment of flow diagram

**Fig 3:**
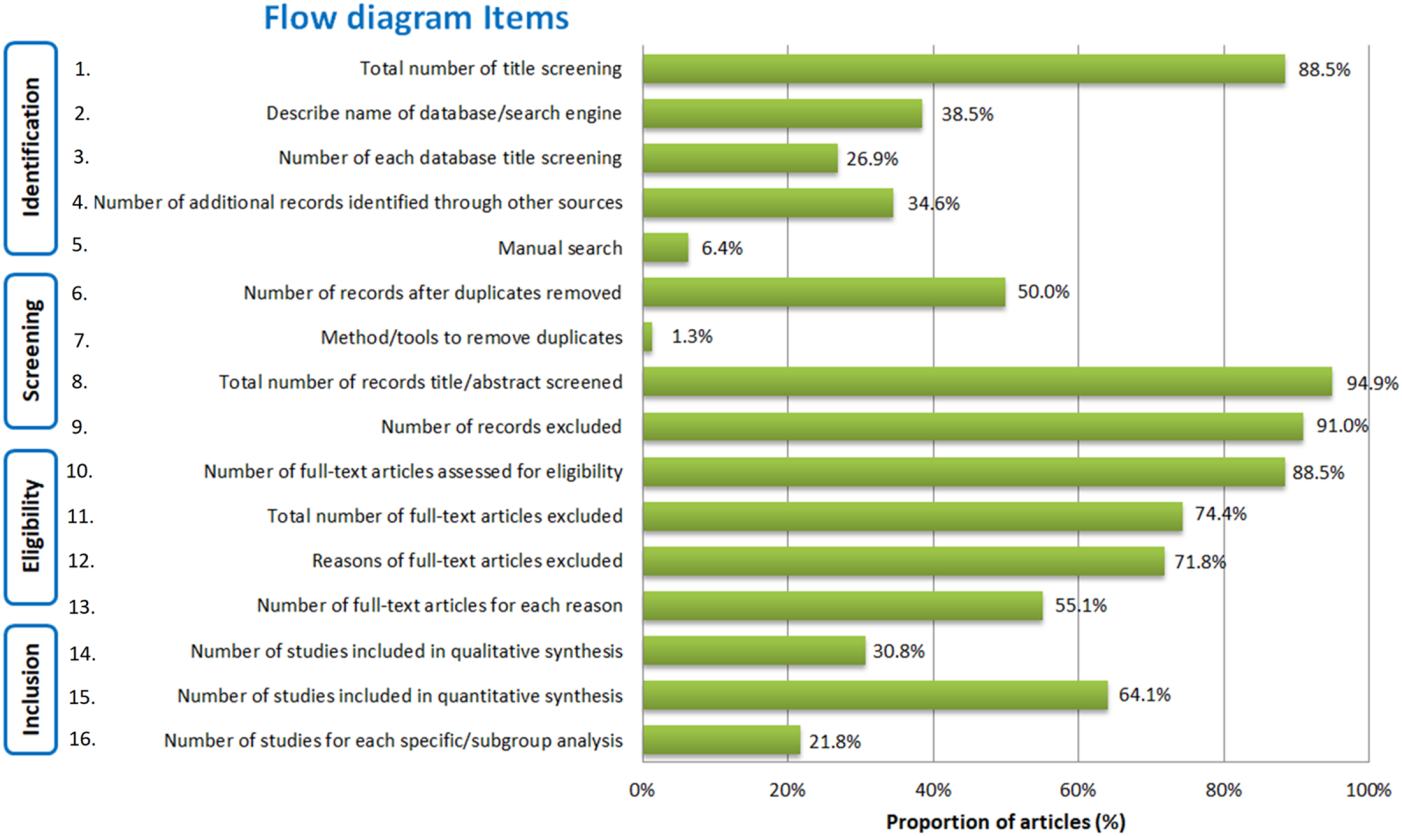
Characteristic of all items in flow diagram and the presence of each item.

Our analysis revealed some variation in the proportion of flow diagrams, which fulfilled different components of the flow diagram checklist. Of all 78 available flow diagrams, the reporting of items in flow diagram was considerably different: 12% of flow diagrams missed the total number of articles identified, 62% of flow diagrams did not describe the name of databases or search engines used, 73% of flow diagrams ignored the number of papers from each database or search engine (Fig 3). For other search, 65% and 94% of flow diagrams did not show the number of additional records identified through other sources and through manual search, respectively. A quarter (26%) of flow diagrams that did not reveal the total number of full-text articles excluded. Furthermore, the exclusion number and reasons for full-text article screening were not shown in 28% and in 45% of flow diagrams, respectively. While only one out of two flow diagrams demonstrated the number of records after duplicates removed, 69%, 36% and 78% of papers missed the number of studies included in qualitative synthesis, quantitative synthesis (meta-analysis) and subgroup analysis, respectively.

#### Association between the quality of flow diagram and the methodological quality of the systematic review/meta-analysis

**Fig 4:**
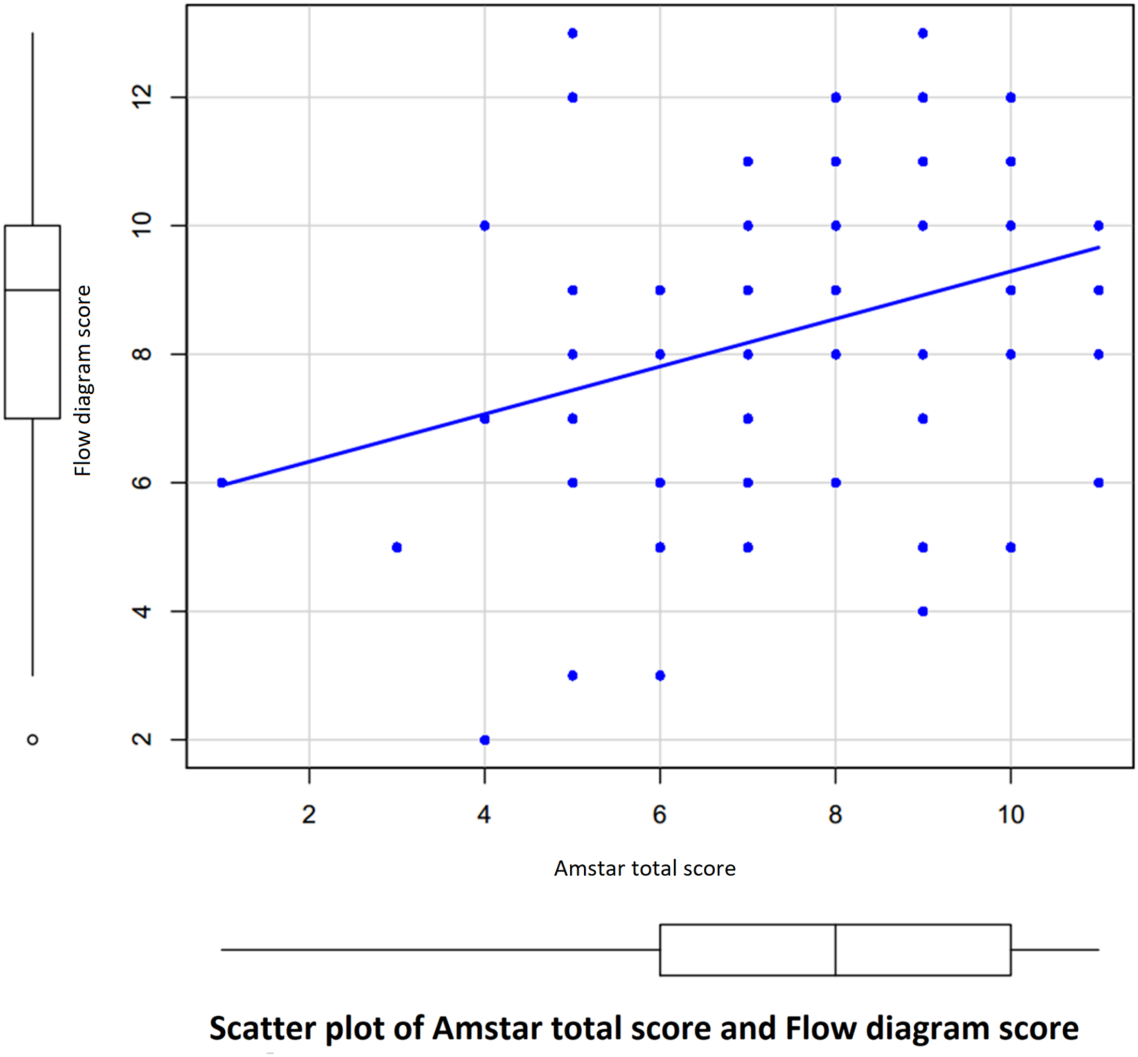
Scatter plot of AMSTAR total score and flow diagram score.

We used the AMSTAR checklist to assess the methodological quality of the systematic review/meta-analysis and found that the AMSTAR total score correlated with flow diagram scores in 78 studies (Pearson correlation coefficient r = 0.32, p = 0.0039) (Fig 4). The mean [standard deviation; SD] of AMSTAR score was significantly higher in flow diagram available group (7.79 [2.19]) than in flow diagram not-available group (6.00 [2.37]); p < 0.001.

**Fig 5:**
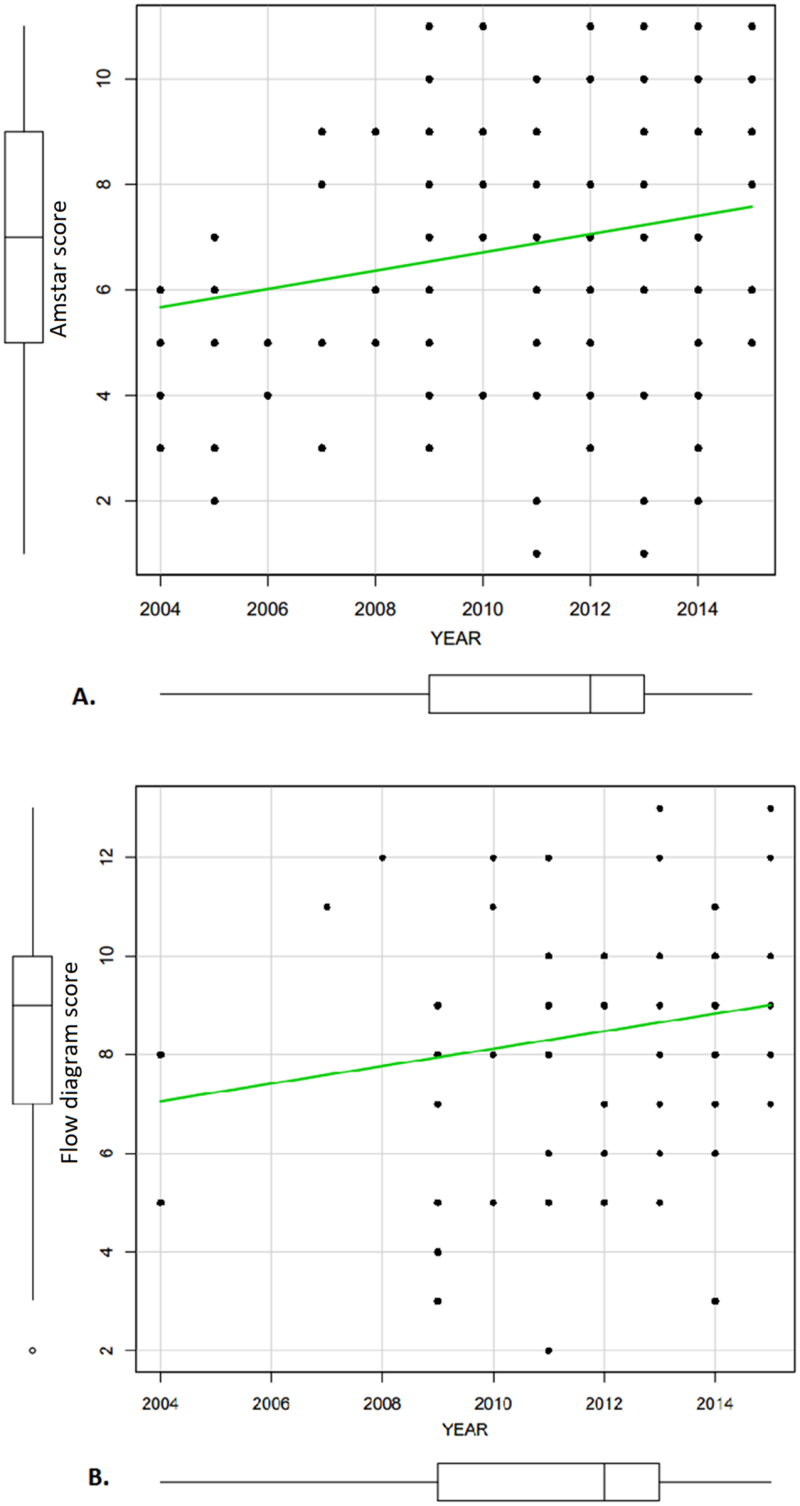
Trend of AMSTAR score (A) and flow diagram score (B) over ten years.

No statistically significant change was observed between the AMSTAR score of articles published before and in 2009 (6.42 [2.34]) and after 2009 (7.10 [2.46]) (p = 0.1149). Over a period of ten years, AMSTAR score had been significantly increasing with the Pearson correlation r = 0.2 (p = 0.012) and the regression coefficient beta = 0.174 (p = 0.012). However, during the same period, flow diagram score had been gradually increasing but not significantly with the Pearson correlation r = 0.172 (p = 0.133) and the regression coefficient beta = 0.177 (p = 0.133) (Fig 5).

#### Association between the quality of flow diagram and the impact factor of journals

**Fig 6:**
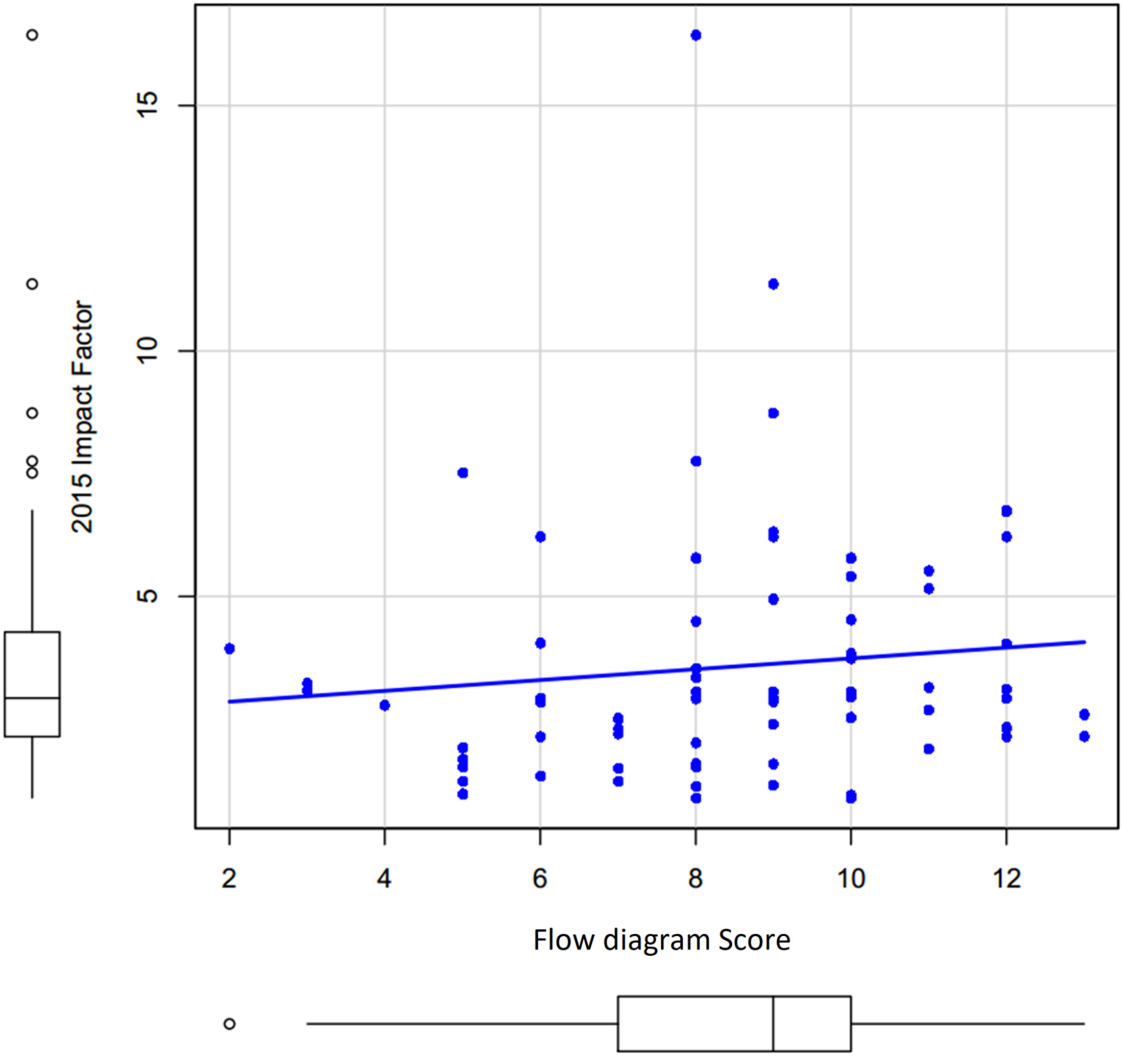
Correlation of flow diagram score and impact factor of journals.

The correlation between flow diagram score and impact factor was not significant with the Spearman correlation r = 0.21 (p = 0.07) and the regression coefficient beta = 0.11 (p = 0.35) (Fig 6).

Our results revealed a higher in the adherence to PRISMA checklist in the group with flow diagram (66.2 ± 17.6%) compared to the group without flow diagram (54.8 ± 19.7%) (Table 1).

**Table 1:** Adherence to PRISMA checklist of 154 systematic reviews and/or meta-analysis in two groups: With flow diagram and without flow diagram.

	Group without flow diagram (N = 76)	Group with flow diagram (N = 78)
**Description of PRISMA item**		
**TITLE**		
Title	50 (65.8%)	64 (82.1%)
**ABSTRACT**		
Structured summary	63 (82.9%)	68 (87.2%)
**INTRODUCTION**		
Rationale	75 (98.7%)	78 (100.0%)
Objectives	74 (97.4%)	73 (93.6%)
**METHODS**		
Protocol and registration	5 (6.6%)	6 (7.7%)
Eligibility criteria	70 (92.1%)	77 (98.7%)
Information sources	71 (93.4%)	77 (98.7%)
Search	58 (76.3%)	68 (87.2%)
Study selection	63 (82.9%)	75 (96.2%)
Data collection process	27 (35.5%)	37 (47.4%)
Data items	33 (43.4%)	38 (48.7%)
Risk of bias in individual studies	23 (30.3%)	37 (47.4%)
Summary measures	38 (50.0%)	46 (59.0%)
Synthesis of results	31 (40.8%)	40 (51.3%)
Risk of bias across studies	19 (25.0%)	24 (30.8%)
Additional analyses	23 (30.3%)	23 (29.5%)
**RESULTS**		
Study selection	30 (39.5%)	75 (96.2%)
Study characteristics	50 (65.8%)	72 (92.3%)
Risk of bias within studies	15 (19.7%)	37 (47.4%)
Results of individual studies	39 (51.3%)	61 (78.2%)
Synthesis of results	34 (44.7%)	43 (55.1%)
Risk of bias across studies	20 (26.3%)	25 (32.1%)
Additional analysis	24 (31.6%)	27 (34.6%)
**DISCUSSION**		
Summary of evidence	54 (71.1%)	66 (84.6%)
Limitations	30 (39.5%)	45 (57.7%)
Conclusions	64 (84.2%)	71 (91.0%)
**FUNDING**		
Funding	42 (55.3%)	42 (53.8%)
**PrismaTotal**	**54.8 (19.7)**	**66.2 (17.6)**

There were eight Cochrane systematic reviews out of 154 included articles. Among 78 articles with flow diagram available, there were three Cochrane systematic reviews. The mean [SD] of flow diagram score was slightly higher in Cochrane group (9.00 [3.00]) than in non-Cochrane group (8.45 [2.51]) but not significantly with p-value = 0.78.

## Discussion

A well reported flow diagram is useful for readers to follow the sequence of the review process and line out any source of bias, such as selection bias [28,29]. Although our current study showed that only 50% of systematic reviews utilized flow diagrams, we do acknowledge that the proportion of papers with flow diagrams available have been significantly increasing over a period of ten years (from 2004 to 2015), suggesting a progressive improvement in methodology of systematic reviews conducted. Our results agree with the study of Daniel Hind et al [17], which also demonstrated that the proportion of systematic reviews containing flow diagram in a 5-year period (from 2001 to 2005) was 49%, and that it was increasing over time. Moreover, more systematic review/meta-analysis published after 2009 (59%) utilized the flow diagram in comparison to papers published before and in 2009 (28%), this might be a plausible supporting of the positive impact and role of PRISMA in the methodological progress of systematic reviews [30–32].

Our assessment of the flow diagrams and its adherence of its components showed that several items of the flow diagram were under reported. The lowest reporting was methods of duplicates removal in screening phase (1%), followed by manual search in identification phase (6%), number of studies for each specific/subgroup analysis (22%), number of articles retrieved from each database (27%), number of studies included for quantitative analysis (31%), number of articles identified from other sources (35%), and description of databases/search engine (39%). In fact, each item plays a certain role in the quality of diagram, so should not be ignored. For instance, the methods or tools to remove duplicates, which were almost under reported, would give a transparent method in removing duplicates, avoid biases and allow readers and reviewers to verify its validity anytime. The manual search, the second item neglected, helps explore more papers which could be missed in other methods, then gives credit to researchers to include enough references to reach accurate results. The next forgotten item is the number of studies for each specific/subgroup analysis which can be responsible for stating the research problem in very specific, definable, and set terms; and for giving an idea about the degree of heterogeneity of the included studies and subsequently the population of the study. The number of each database title screening, in turn, can suggest the biggest databases and maybe the most important databases relating to research topic [27], etc. That also figures out the paramount importance of applying a flow diagram containing full of items—what we did at the end of this article.

Our analysis also highlights that although flow diagram utilization was correlated with the methodological quality of systematic review, it was not associated with the impact factor of journals where they are published. This finding suggests an evaluation of the impact and the improvement in the quality of a good flow diagram would make, given the lack of guideline for flow diagram reporting [33].

If we split the data into two groups: Cochrane and non-Cochrane, the flow diagram score was slightly higher in Cochrane group (9.00 [3.00]) than in non-Cochrane group (8.45 [2.51]) but not significantly (p-value = 0.78) probably due to the small sample size in Cochrane group (n = 3). While, Page and colleagues examined the reports of 300 systematic reviews published in February 2014 and noted important differences between Cochrane and non Cochrane reports including flow diagrams [19].

One limitation in our study is that, although a training testing was conducted prior to completing the AMSTAR assessments, flow diagram quality assessment and PRISMA assessment, we did not compare the assessment before and after training. Moreover, we did not evaluate the other systematic review guidelines, and hence could not compare the effectiveness and limitations among different guidelines, such as MOOSE [34,35]. These guidelines could be important in the improvement of reporting of systematic reviews/meta-analysis [36–41]. The second limitation is that there were three journals with unidentified impact factor given their recent release, e.g. in 2014 or 2015.

**Fig 7:**
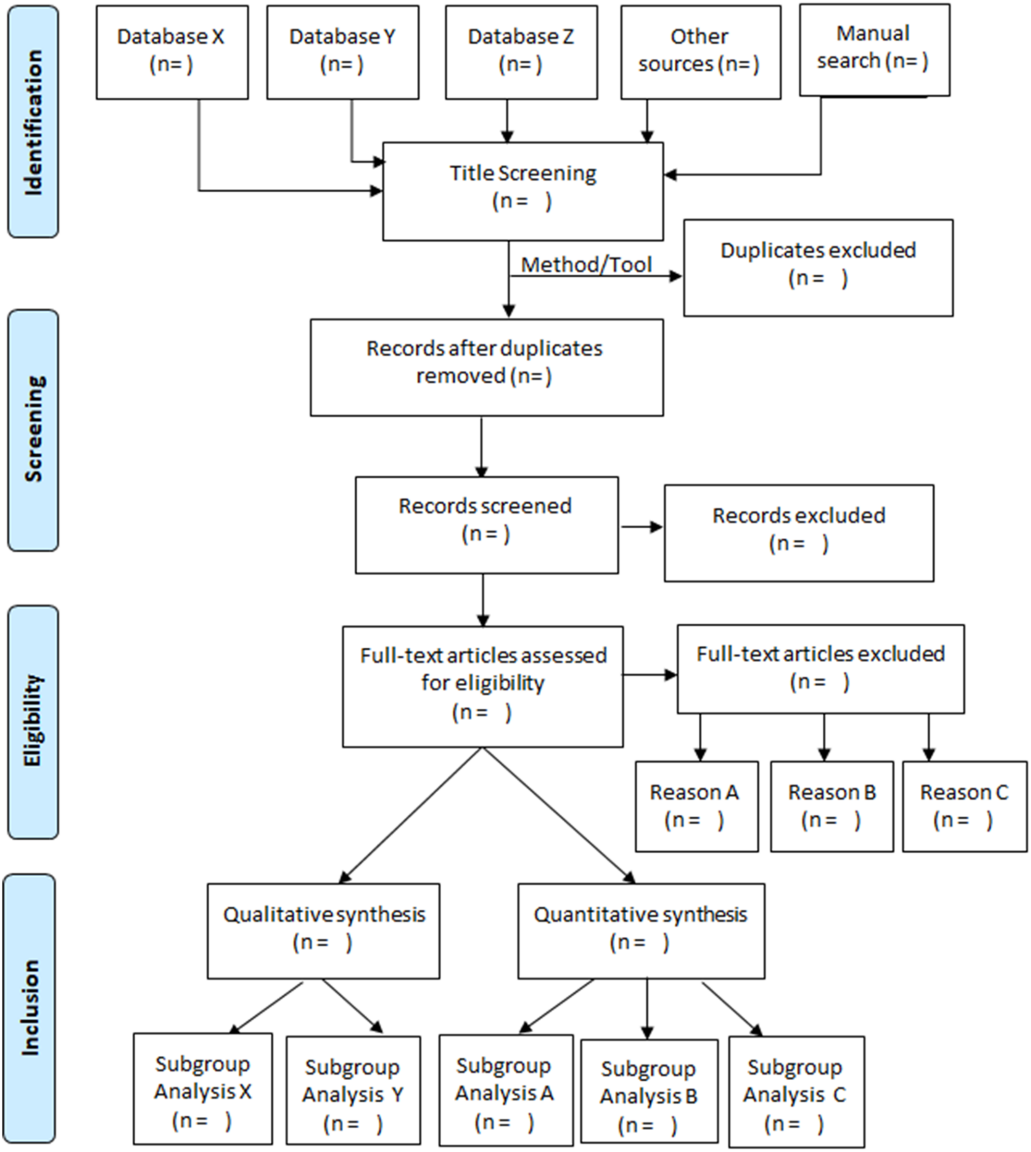
New flow diagram template proposed.

On the other hand, this study is considered to be the first systematic literature review to evaluate the quality and the reporting of flow diagrams ever since the PRISMA statement was released in 2009. In addition, it provides statistical evidence about its relation to the quality of the whole review. Based on the positive correlation of the quality of flow diagram and quality of the whole review, a standardized flow diagram is essential, and should be recommended in all systematic reviews/meta-analysis in order to improve the quality and reliability [14,15]. Therefore, we propose a template of flow diagram containing the full items corresponding to four stages of PRISMA statement (Fig 7).

We committed to the four stages of PRISMA flow diagram (S1 Fig), then we added some items to every stage which helps to provide a better detailed diagram. Specifically, the item “Records identified through database searching” is based on PRISMA flow diagram and clarified by pointing out record from each database. In item “other sources” derived from PRISMA, we developed “other sources” and “manual search”. Then, before item “records after duplicates removed” we put “the number of all records obtained from all databases”. We think that the method/tool to remove duplicates is also important to be included in flow diagram, which helps readers to verify its validity; hence this item is added in our proposed flow diagram. At the bottom of flow diagram which represents the inclusion step, the number of studies for each specific subgroup should be added so that readers can follow more easily the process of research.

## Conclusion

In conclusion, we found that only half of the systematic reviews/meta-analysis presented flow diagrams. The total quality and reporting components in the flow diagram were less than satisfactory, and have not improved over ten years. Our findings indicate that researchers need to improve on their efforts to help readers achieve a comprehensive understanding of the review process.

## Supporting information

S1 Table AMSTAR checklist. (DOCX)

S2 Table Flow diagram items. (DOCX)

S1 Fig PRISMA flow diagram. (TIF)

S2 Fig The proportion of papers with flow diagram over five years (Daniel Hind et al 2007). (TIF)
