# Supplementary material for: Quality of flow diagram in systematic review and/or meta-analysis
Source: PLoS One. 2018 Jun 27;13(6):e0195955. doi: 10.1371/journal.pone.0195955 (PMC6021048; doi:10.1371/journal.pone.0195955)
Supplement: S2 Table — (DOCX) [file pone.0195955.s002.docx]

| **Stage** | **Flow diagram items** |
| --- | --- |
| **① Identification** | Total number of title screening |
|  | Describe name of database/search engine |
|  | Number of each database title screening |
|  | Number of additional records identified through other sources |
|  | Manual search |
| **② Screening** | Number of records after duplicates removed |
|  | Method/tools to remove duplicates |
|  | Total number of records title/abstract screened |
|  | Number of records excluded |
| **③ Eligibility** | Number of full-text articles assessed for eligibility |
|  | Total number of full-text articles excluded |
|  | Reasons of full-text articles excluded |
|  | Number of full-text articles for each reason |
| **④ Inclusion** | Number of studies included in qualitative synthesis |
|  | Number of studies included in quantitative synthesis |
|  | Number of studies for each specific/subgroup analysis |
