# Supplementary figures and images for: Quality of flow diagram in systematic review and/or meta-analysis
Source: PLoS One. 2018 Jun 27;13(6):e0195955. doi: 10.1371/journal.pone.0195955 (PMC6021048; doi:10.1371/journal.pone.0195955)

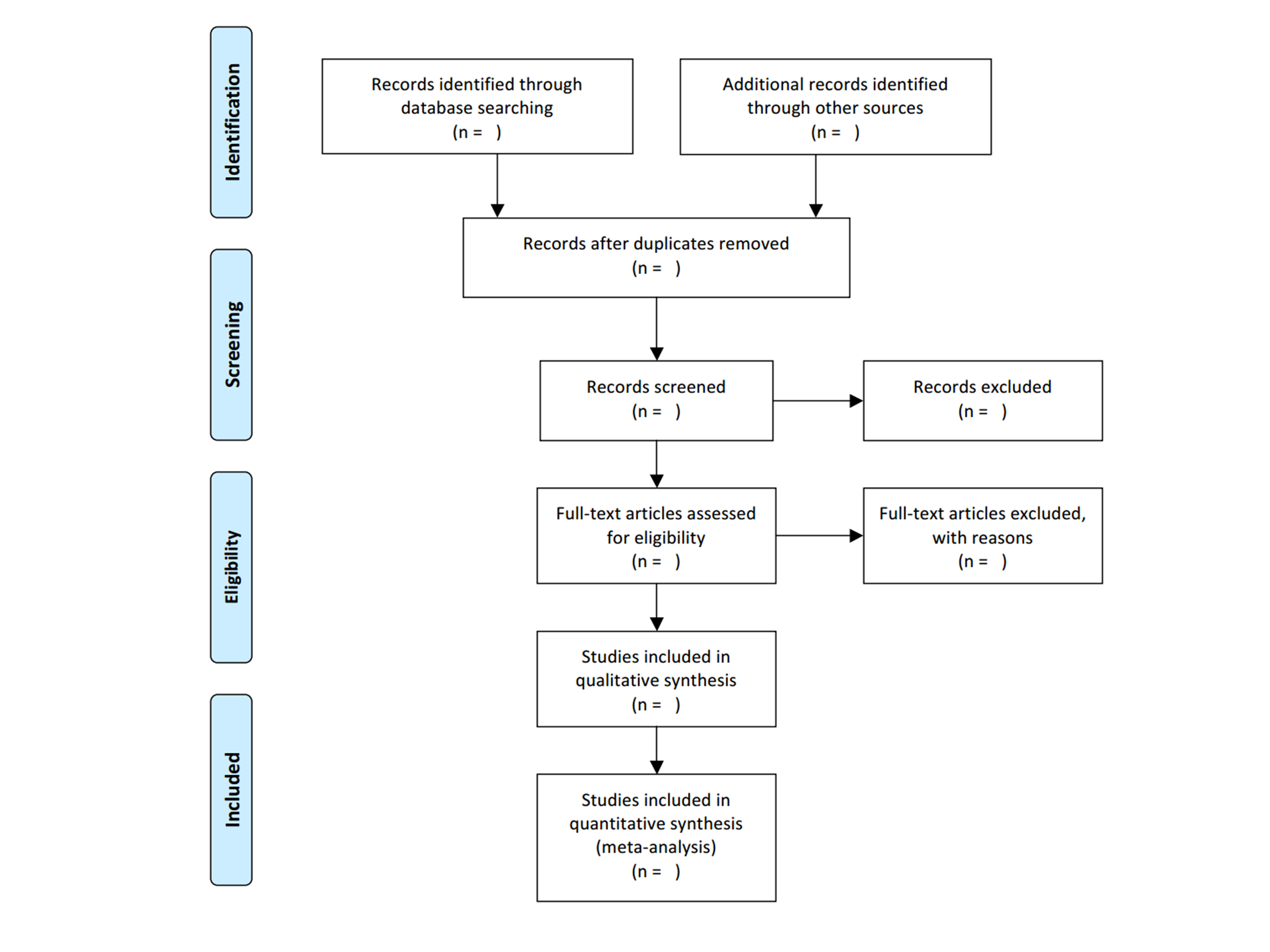

Supplement: S1 Fig — (TIF) [file pone.0195955.s003.tif]

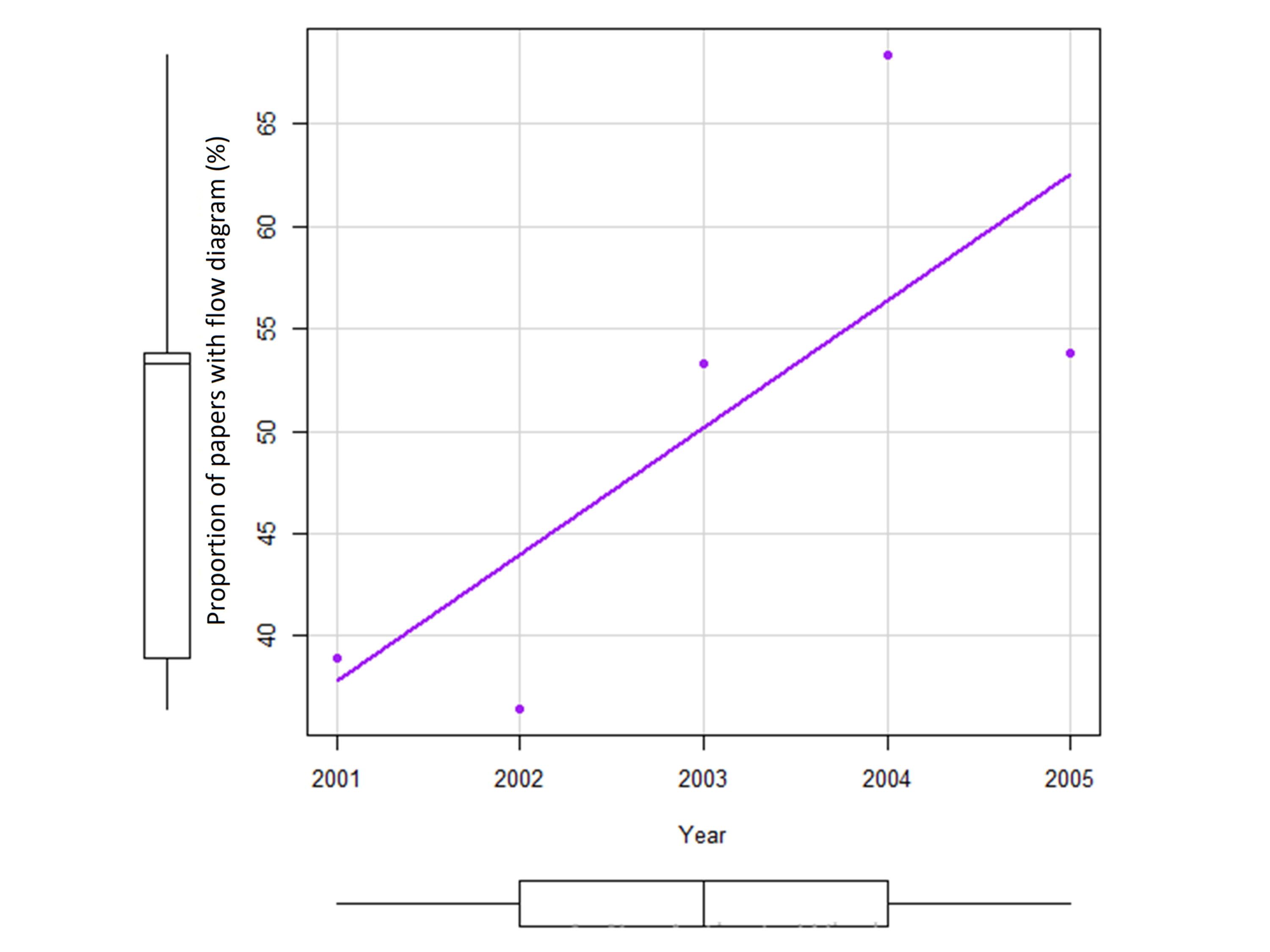

Supplement: S2 Fig — (TIF) [file pone.0195955.s004.tif]
